# Foreign

**Published:** 1842-01-29

**Authors:** 


### FOREIGN.

*Anchylosis of the Lower Maxilla with the Temporal Bones.*—Dr. Payan, Surgeon to the Hôtel Dieu at Aix, in a collection of facts on different diseases of the head, which he has just published, reports this remarkable case. It seems that the patient, 77 years of age, was attacked by the cholera morbus in 1835, and carried to the hospital, where he died in two days. It was observed upon his admission that he could not move the lower jaw, and that the tongue was protruded through an aperture formed by the loss of the four upper incisor teeth. His children and relations stated, that during his lifetime he had frequently told them that he had been unable to move his jaw from between four and five years of age, which he attributed to an accident he met with at that time, viz. the fall of a table upon his head, probably causing fracture of the maxilla near the condyles. Upon dissection, complete ossification was found to have taken place with both temporal bones, and the union was so perfect that no line of demarcation could be seen between them. All the teeth existed, with the exception of one or two molar, and the four upper incisors which had been extracted to admit the introduction of food. They were in close apposition with each other. The inferior incisors were pushed more forwards, and the molars more outwards, than in the natural state. In each parotid duct was found a calculus. No trace of fracture could be discovered in any part of the bone. There was no anchylosis of any other joint. This curious specimen has been frequently exhibited by M. Dubreuil to the medical students at Montpelier.

Cases of anchylosis of this articulation have been recorded before, but they have generally been found only when other joints had undergone the process. An officer died at Metz in 1803, who had long been affected with general rheumatism, brought on by fatigue during the war, in a cold and moist country. Upon dissection, every articulation was found anchylosed, and his skeleton, which is now in the museum of l'Ecole de Médecine, forms in reality a single bone.—*Lond. and Edin. Monthly Journ. of Med Sci.*, from *Gaz. Med. de Paris*, Aug. 28, 1841.

---

*Case of Perforation of the Stomach.* By John Moyle, Esq.—On Wednesday, the 20th, ult., at 4 P. M., I was requested to visit Mary M., aged seventeen years. About an hour previous to my seeing her, she had been seized with severe pains in the stomach and abdomen, (shooting to the right shoulder,) aggravated in paroxysms and on pressure; the pulse was extremely quick, small, and depressed; extremities warm; the countenance expressive of hopeless anxiety; and the stomach occasionally rejected its contents. Bleeding, (to ℥iij. only,) instead of being followed by the satisfactory rise of the pulse which we look for in ordinary cases of membranous inflammation, caused only greater rapidity and further exhaustion. Her bowels had not been opened since the 25th. Warm fomentations, mustard poultices, purgatives, creosote, and turpentine injections gave little or no relief.

30th, 8 A. M.—She had passed a very bad night as to pain; she had voided only a very small quantity of high-coloured urine; tongue clean; skin pale; no medicine remains on her stomach; bowels have not been relieved by the injections. She had been subject to pains in the stomach for four or five years, and had never menstruated; could lie on her back only: there was no hernia in any of the usual situations.

5½ P. M.—Pain still very severe; distressing vomiting and urgent thirst; bowels not yet open; extremities cold. From the moment of the seizure to its fatal termination no remedy gave relief, and in twenty-six hours the poor girl was released from intense suffering.

*Post-mortem examination, eighteen hours after death.*—The body, which inclined to *embonpoint*, presented no mark of disease externally, excepting a tympanitic state of the abdomen, which had commenced slightly before death.

The abdominal cavity contained a considerable quantity of air, and about three pints of dirty yellowish fluid. The intestines were slightly streaked with red, and their walls were slightly adherent together by soft recently effused lymph. This peritonitis was found to have been caused by the effusion of the contents of the stomach through an ulcerated aperture in its walls. The ulcer was circular, about three-eighths of an inch in diameter, with hardened edges. It was situated about two inches and a half to the right of the œsophageal opening on its anterior wall; the cellular tissue, for about three inches in length, and one and a quarter in breadth, was thickened, brawny, and puckered like a cicatrix. The internal surface of the stomach presented a funnel-shaped ulcer through its coats, the size of a shilling, without any surrounding inflammatory blush: there was a second ulcer about two inches below the first, of greater size, but of the same character, effusion being prevented only by its peritoneal covering. The liver was small, very white, and bloodless when cut into; and its texture easily broken down with the finger; the gall-bladder contained about an ounce and a half of thin and very light-coloured bile. In every other respect the abdominal viscera (I was not allowed to open the chest) presented the appearance of perfect and very robust health.

The only novelty which this case possesses is the absence of pain "*between the shoulders*," which has been considered, by Dr. Abercrombie and others, as practically diagnostic.

It is also confirmatory (if further proof were wanting) of the extent to which disease of the cardiac extremity of the stomach may proceed, without apparent loss of health.—*London Medical Gazette*, October 15, 1841.

---

*Researches into the real constitution of the Atmosphere.* By MM. Dumas and Boussaingault.—It is generally admitted that the air is composed of a mixture of oxygen and nitrogen, and its invariableness is explained by supposing that the green parts of plants under the influence of solar light decompose all the carbonic acid developed in the respiration of animals, and the putrefaction of organized bodies. Some, however, regard the air as being not a mixture, but a chemical compound of 20 of oxygen and 80 of nitrogen, (Prout, Dobereiner, Thomson, &c.) Others, and these the majority, consider it as a mixture of 21 of oxygen and 29 of nitrogen; and, lastly, in the opinion of some, (Dalton, Babinet,) the composition of the air varies according to the height in the atmosphere.

The plan employed by the authors in submitting these questions to a fresh examination is distinguished from others in that they estimate the weight instead of the measure of the gases, and thus analyze the air by weighing successively the oxygen and the nitrogen which it contains. We cannot follow them into all the details of their experiments, which, by successive corrections, were rendered more and more exact: we can only point out the results.

They fix the density of oxygen at 1.1057, and that of nitrogen at 0.972; numbers a little different from those given by other chemists. They demonstrate that the relation of the volume of the oxygen to that of the nitrogen in the air is not expressed by simple numbers; and that the air cannot be regarded as a chemical composed of 20 volumes of oxygen and 80 of nitrogen. They admit, as *a sufficient approximation*, that the atmosphere is composed, by volume, of 20.8 of oxygen, and 79.2 of nitrogen. They presume that the mixture is uniform in all times, in every latitude, and at every height. "If the atmospheric air," they add, "is a reservoir of oxygen for the use of animals, and a reservoir of carbonic acid for the use of plants, it is so considerable a store that the consumption, supposing it not to be compensated, would remain almost insensible after a long series of years." They have calculated that supposing each man to consume a kilogramme of oxygen per day, and that the oxgen disengaged by plants did no more than compensate for the other causes of its absorption, the whole human race, and three times their number, would not consume, in a century, the eight-thousandth part of the oxygen which nature has placed in the respirable air—*Lond. Med. Gaz.*, Oct. 15, 1841, *from L'Examinateur Medical,* Aout 20, 1841.

---

*On the Influence of the last Inundation on the health of the Population of Lyons.*—It was a matter of much interest to determine what were the effects on the health of its inhabitants of the inundation, which, in so short a time, on two different occasions, covered a part of the town of Lyons. If mere variations of temperature and atmosphereic moisture do exercise on the health only a part of the fatal effects attributed to them, it is certainly, in a case like that of Lyons, that such effects ought to be easily appreciable. In no inundation of that town had the waters ever before risen to such a height: in none was their stay so long, or their retreat so slow. They covered the town and the country round over an area of several leagues: they inundated the quays, the streets, the places of public resort the cellars, the houses; and they polluted the waters of the wells, the pumps, and springs, with heterogeneous substances that filtered into them from the sewers and the privies. After their retreat, a stinking sediment covered the majority of the streets, and a thick viscid mould lined the inner walls of the houses: the atmosphere was charged with a nauseous dampness: the population was distressed: every thing was dreary; and yet the inundation of 1840 had but a just perceptible influence upon the public health. From the month of November, in that year to the present time (July, 1841,) the hospitals have not been more than usually encumbered: the numbers of patients has been but little increased; and, though the mortality has been distinctly greater, yet this cannot be attributed to any disease in particular produced by the inundation.

In the days immediately following this scourge, a considerable number of cases of obstinate diarrhœa, and some of dysentery attributed to drinking the unwholesome water, were observed. Some typhoid fevers also appeared; and, if the state of the atmosphere had any influence upon them, it was only by prolonging their continuance, and giving them a more marked adynamic character. Still they were not more fatal than they usually are, though it was necessary to modify the ordinary treatment; blood-lettings at the outset were less employed, and tonics were sooner and longer administered.

At a later period numerous and obstinate rheumatisms were observed, but they rarely had an acute character, except in those who had worked long in the water: in general the moisture of the atmosphere rather revived old pains than generated new ones. The diseases which reigned most generally, were catarrhs and catarrhal fevers, which, however, are habitually prevalent at Lyons at this season; but the inundation remarkably increased their number and modified their character; some affecting the lungs, had an alarming intensity and obstinacy, but on the whole they destroyed but few. An epidemic disease, of which both the etymology and etiology are still obscure, reigned at this time at the garrison, but it could not be, in any measure, traced to the effects of the inundation.

Notwithstanding the absence of any special affection, however, the mortality was more considerable than during the preceding years. Thus, in the last two months of 1840, the town and hospitals together had 1118 deaths; while in the same two months of 1839, there were but 787, and of 1838 only 750. But this difference is easily explained by the pernicious influence such a scourge would have on aged, weak, and diseased people, and especially on such as were already affected with chronic diseases.

The author attributes the good state of health of the town and its environs, during the continuance of the scourge, to the prompt and energetic measures, which were taken by the local administration, and to the north wind, which continued for twenty days, and greatly aided the sanitary measures that were being employed.—*Lond. Med. Gaz. November* 5, 1841, *from Journal de Medecine de Lyons*, Juillet, 1841.

*M. Raciborski on the Physiology of Menstruation.*—Our readers will remember that in our recent review of Gendrin's *Traite Philosophique,* we drew their attention to this curious, and hitherto ill-understood subject of physiological inquiry. From the researches of this gentleman, and from those of M. Negrier, as well as of Dr. Robert Lee, it was suggested that there is an actual rupture of one of the ovarian vesicles at each period of menstruation, and that the sanguineous discharge from the uterus was the result of this lesion. M. Raciborski questions the accuracy of this statement. While he admits that the primary movement in each act of menstruation is a congestion of the vessels of the ovaries, he denies that any rupture of their surface necessarily takes place at the same time.

M. Raciborski sums up the conclusions to which he has come, after a very elaborate inquiry, in the following propositions:—

1. That menstruation is a consequence of the accomplishment of the development of the ovaries.

2. That it is the direct result of the means employed by nature to place the ends of the Fallopian tubes and the ovaries in the relations necessary to fecundation and the passage of the fecundated ova.

3. That the sanguineous congestion, which is indispensable for obtaining those conditions in the human being, appears sufficient in itself to explain the occurrence of the hæmorrhage which constitutes menstruation—without having recourse to supposing that there is any necessary solution of continuity.

4. That the vertical position, favouring still more the effects of sanguineous congestion on the generative organs, may be one of the principal reasons of the abundance of the menstrual flux in women, and in some species of simiæ.

5. That, for want of having precise information as to the nature and theory of menstruation, it has been hitherto impossible to establish a rational treatment of the various disorders induced by irregularities of this function.

6. That it is not yet sufficiently proved that the ovula arrive successively to maturity at each menstrual epoch, or that the most mature ovum then approaches nearest the surface of the ovarium, there to become ruptured and give exit to a germ.—*Med. Chir. Rev.* Oct. 1841, *from L'Experience.*

